# Effect of Diazepam on Severity of Acute Pancreatitis: Possible Involvement of Peripheral Benzodiazepine Receptors

**DOI:** 10.1155/2013/484128

**Published:** 2013-07-17

**Authors:** Alireza Abed, Mohsen Minaiyan, Azadeh Safaei, Diana Taheri

**Affiliations:** ^1^Department of Pharmacology and Toxicology, School of Pharmacy and Pharmaceutical Sciences, Isfahan University of Medical Sciences, Isfahan 8146-73461, Iran; ^2^Department of Pharmacology and Toxicology, School of Pharmacy and Pharmaceutical Sciences and Isfahan Pharmaceutical Sciences Research Center, Isfahan University of Medical Sciences, Isfahan 8146-73461, Iran; ^3^Department of Clinical Pathology, School of Medicine, Isfahan University of Medical Sciences, Isfahan 8146-73461, Iran

## Abstract

Acute pancreatitis is a lethal inflammatory condition of pancreas with high mortality rate. There is a pressing need for research to explore active agents and novel mechanisms involving in the treatment of pancreatitis. Clinical studies have shown after the initial acinar cell injury plasma levels of pro-inflammatory cytokines are elevated in patients with acute pancreatitis and the degree of cytokine elevation correlates with disease severity. Diazepam may decrease interleukin release from macrophages, suppress neutrophil activities, and exhibit anti-inflammatory effects. So it is expected that *in vivo* pretreatment of acute pancreatitis with different doses of diazepam can attenuate its severity. Thus, we evaluated the effects of diazepam, intraperitoneally (5, 10, and 20 mg/kg i.p.), intracerebroventricularly (ICV 10 **μ**g), and concurrently with flumazenil (1 mg/kg) on cerulein-induced acute pancreatitis in mice. Interestingly, the pretreatment with diazepam (5 mg/kg i.p.) reduced significantly the inflammatory response of acute pancreatitis by ameliorating pancreatic edema, amylase and lipase serum levels, myeloperoxidase activity, pancreatic TNF-alpha, and pathological alteration compared to control group. Diazepam i.c.v. was ineffective, suggesting that central benzodiazepine receptors have no significant role in this property. These results demonstrate that pretreatment with diazepam exhibits anti-inflammatory property in cerulein-induced acute pancreatitis possibly through peripheral benzodiazepine receptors.

## 1. Introduction

Acute pancreatitis is a potentially lethal disorder which is characterized by pancreatic tissue inflammation and elevation in serum level of digestive enzymes without specific therapy [1]. The morbidity and mortality associated with pancreatitis are secondary to circulatory shock, cardiac insufficiency, respiratory distress, and hepatic failure [2]. The main causes of pancreatitis are alcohol beverages drinking and biliary tract disorders which are the most important etiologies of pancreatitis [3].

The early pathophysiology of the acute pancreatitis has not been well understood [4]. But some clinical studies have shown that after an initial acinar cell injury, proinflammatory cytokines such as interleukin-(IL-) 1, tumor necrosis factor, IL-6, and IL-8 are increased in the serum of patients with acute pancreatitis, and the degree of cytokine elevation correlates with disease severity [5]. Cytokines are responsible for both the local and the systemic inflammatory response and organ dysfunction in acute pancreatitis [6]. Also, a number of animal studies showed the role of proinflammatory cytokines in pancreatitis severity which is predictive for mortality [7]. New therapeutic approaches aim to modulate these pathways.

Diazepam is a classical benzodiazepine (BDZ) with sedative, anxiolytic, and anticonvulsant properties. It has been found that diazepam binds with high affinity to peripheral benzodiazepine receptors (PBRs) which are distinct from the central benzodiazepine receptors (CBRs) [8]. PBRs are which known as translocator protein, are widely distributed transmembrane proteins that are located mainly in the outer mitochondrial membrane. PBRs have been found in many organs such as the pancreas, lungs, kidneys, liver, adrenals, and also in immune cells [9].

It has been shown that acute treatment with diazepam decreases interleukin release from macrophages and suppresses neutrophil activities [10]. Some studies have been shown that high doses of diazepam (10 and 20 mg/kg) suppressed the inflammation in rats because of the presence of PBRs in the adrenal and immune cells [11]. Also it has been found that acute treatment with diazepam increases plasma level of corticosterone which is the main glucocorticoid, via acting on PBRs on adrenal gland [12]. Recently, it has been demonstrated that endogenous glucocorticoids are an important factor for acinar cell survival. Endogenous glucocorticoids protect acinar cells by decreasing their sensitivity to the induction of cell death during acute pancreatitis [13]. 

It also has been determined that treatment of mice with PBRs agonist Ro5-4864 markedly reduces the production of inflammatory mediators such as interleukin-1, TNF-*α*, and interleukin-6 by macrophages [14]. 

These data suggest that diazepam which acts on PBR as well as CBRs might possess protective effects against pancreatitis.

The present study was performed to evaluate the protective effects of diazepam in an animal model of cerulein-induced acute pancreatitis in mice. To gain access to better insight into the mechanism(s) of actions of diazepam on pancreatitis, we have investigated the effects of diazepam administration, both peripherally and centrally, on pancreatic edema, leukocyte infiltration, amylase and lipase levels, TNF-alpha, and myeloperoxidase activity.

## 2. Materials and Methods

### 2.1. Animals

Male mice weighting 25–30 g and bred in animal house (Isfahan School of Pharmacy, Isfahan, Iran) were used in this study. Animals were kept in uniform environment of temperature, humidity, and light/dark cycles (12/12 h) and allowed free access to pelleted rodent chow and tap water. Before initiation, the experiment animals were fasted over the night. The study was approved by the Ethics Committee for Animal Care and Uses, Isfahan University of Medical Sciences, Isfahan, Iran.

### 2.2. Surgical Procedures

Mice were anesthetized with i.p. injection of a ketamine (50 mg/kg) and xylazine (10 mg/kg) mixture and positioned in a stereotaxic instrument (Stoelting, USA). The guide cannula was positioned at stereotaxic coordinates of AP: −0.5 mm, relative to bregma; lateral: 0.8 mm; dorsal-ventral: −2.5 mm. At least seven days were allowed for recovery after the implantation of the ICV guide cannula before experimental protocols were begun. The injection sites were verified by injecting the same volume (2 *μ*L) of 1% methylene blue into the site and then observing the distribution of the injected dye in the ventricular space. The dye-injected i.c.v. was found to be distributed in the ventricular spaces and ventral surface of the brain and in the upper cervical portion of the spinal cord [15].

### 2.3. Induction of Pancreatitis

Acute pancreatitis was induced by five intraperitoneal (i.p.) injection of 50 *μ*g/kg body weight of cerulein (Sigma, St. Louis, MO, USA) with 1 h intervals according to the method which was previously demonstrated by Mazzon et al. [16]. Cerulein induced acute pancreatitis with prominent rising in amylase and lipase level and increasing TNF-alpha and myeloperoxidase (MPO) activity. 

### 2.4. Experimental Design

In the first series of experiments, effect of i.p. diazepam (5, 10, and 20 mg/kg, *n* = 6) on acute pancreatitis was studied. Diazepam was given 15 min before induction of pancreatitis. 

Control group received only vehicle (i.p.; *n* = 6). In negative control groups, mice with acute pancreatitis were pretreated with normal saline (5 mL/kg and i.p).

In the second series, diazepam (20 mg/kg i.p.) was administered with flumazenil (1 mg/kg i.p.) for blocking the central effect of diazepam. 

In the third series, to distinguish between the central and peripheral effects of diazepam we also evaluated the supraspinal administration of diazepam (i.c.v) in pancreatitis. The drug was administered smoothly for 1 min through the injection cannula (10 *μ*g/mouse, *n* = 6) 15 min prior to induction of acute pancreatitis.

The control group received vehicle (i.c.v.; 2 *μ*L; *n* = 6). 

Mice were sacrificed 6 h after the last injection of cerulein. Blood samples were attained by directed intracardiac puncture and stored at −70° for biochemical analysis. After decapitation, the pancreas was rapidly removed and fixed in formaldehyde (10%) for histological examination. Besides, portions of this organ were promptly frozen in liquid nitrogen and stored at −70°C until assayed.

### 2.5. Amylase and Lipase Serum Level Analysis

Serum lipase and amylase activity were determined by using commercially available lipase and amylase kits (Pars-Azmoon Company, Tehran, Iran) [17].

### 2.6. Myeloperoxidase Activity Assay

MPO activity, an index of polymorphonuclear cell accumulation, was measured according to the modified method of Bradley et al., pancreas tissue was homogenized in 1 mL of 50 mM potassium phosphate buffer containing 0.5% HTAB (hexadecyltrimethylammonium bromide). Then, the homogenate was sonicated in an ice bath for 10 s, freeze thawed thrice with sonication between cycles. After that, the suspensions were centrifuged at 15,000 rpm for 15 min at 4°C and then the supernatant (0.1 mL) was allowed to react with 2.9 mL of 50 mM potassium phosphate buffer (pH 6.0) containing O-dianisidine dihydrochloride (0.167 mg/mL) and 0.005% hydrogen peroxide. The absorbance of the reaction mixture was measured at 450 nm using a UV-Vis spectrophotometer. MPO activity was expressed in units (U) per gram of wet tissue weight [18].

### 2.7. Measurement of Pancreatic Cytokines

Tissue TNF-alpha was measured using an enzyme-linked immunosorbent assay (ELISA) commercial kit according to the manufacturer's instructions (TNF-*α* ELISA kit Glory Science Co., Ltd., Hong Kong). The cytokine levels were calculated after plotting the standard curves and expressed as pg/mL.

### 2.8. Histological Examination

Paraffin-embedded pancreas samples were sectioned (5 *μ*m), stained with hematoxylin and eosin (H&E), and examined by coworker pathologist unaware of experimental protocol.

The histological grading of edema was made using a scale ranging from 0 to 3 (0 = no edema, 1 = interlobular edema, 2 = interlobular and moderate intralobular edema, and 3 = interlobular edema and severe intralobular edema). Leukocyte infiltration was also graded from 0 to 3 (0 = absent, 1 = scarce perivascular infiltration, 2 = moderate perivascular and scarce diffuse infiltration, and 3 = abundant diffuse infiltration) [19].

### 2.9. Statistical Analysis

Biochemical results are expressed as mean ± SEM. Statistical analysis was carried out by one-way analysis of variance (ANOVA) followed by Tukey's multiple comparison test. Nonparametric data was analyzed by Mann-Whitney *U* test. The minimal level of significance was considered at *P* < 0.05.

## 3. Results

### 3.1. Effects of Diazepam on the Serum Levels of Amylase and Lipase

**Figure 1:**
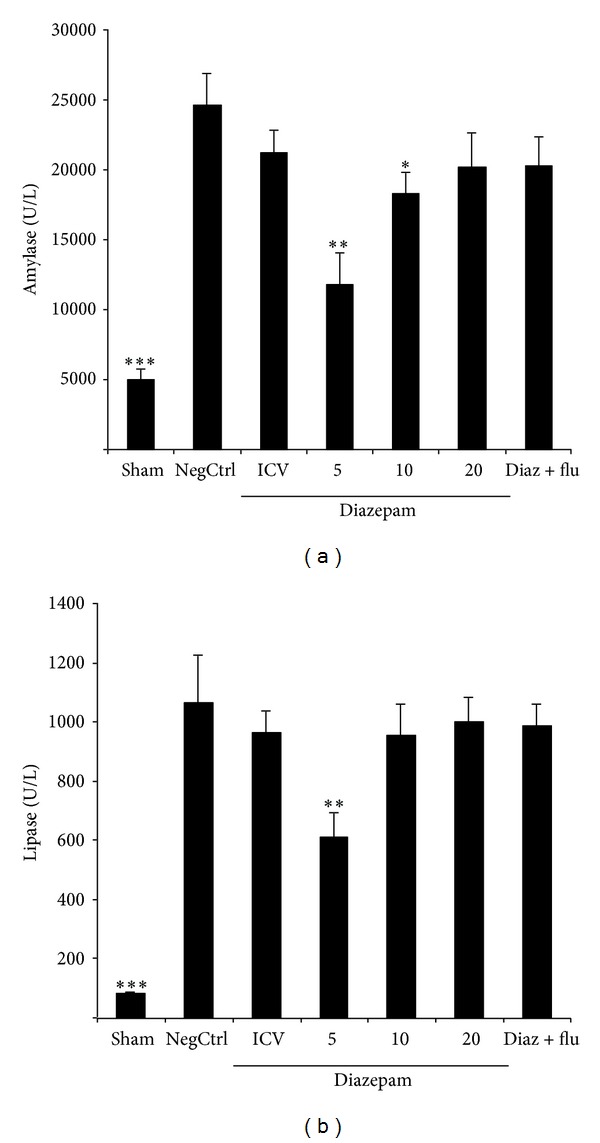
(a) Effect of diazepam on serum amylase level (U/L) of cerulein-induced acute pancreatitis in mice. Sham: normal mice treated with normal saline (5 mL/kg i.p. and 2 *μ*L i.c.v), NegCtrl: mice with pancreatitis treated with normal saline (5 mL/kg i.p. and 2 *μ*L i.c.v.), Diazepam: treated mice (5, 10, and 20 mg/kg i.p. and 10 *μ*g i.c.v.), Diaz + flu: diazepam (20 mg/kg i.p.) + flumazenil (1 mg/kg i.p.). Data are shown as means ± SEM of 6 animals for each group. *P < 0.05, **P < 0.01, ***P < 0.001 versus negative control (ANOVA). (b) Effect of diazepam on serum lipase level (U/L) of cerulein-induced acute pancreatitis in mice. Sham: normal mice treated with normal saline (5 mL/kg i.p. and 2 *μ*L i.c.v), NegCtrl: negative control, mice with pancreatitis treated with normal saline (5 mL/kg i.p. and 2 *μ* i.c.v.), Diazepam: treated mice (5, 10, and 20 mg/kg i.p. and 10 *μ*g i.c.v), Diaz + flu: diazepam (20 mg/kg i.p.) + flumazenil (1 mg/kg i.p.). Data are shown as means ± SEM of 6 animals for each group. **P < 0.01, ***P < 0.001 versus negative control (ANOVA).

Cerulein-induced pancreatitis in vehicle-treated mice was associated with significant rises in the serum levels of amylase and lipase. The increase in amylase and lipase was noticeably reduced in cerulein-treated mice which had been pretreated with diazepam in doses of 5 and 10 mg/kg by i.p. injection (Figures 1(a) and 1(b)).

### 3.2. Effects of Diazepam on Production of TNF-Alpha

**Figure 2:**
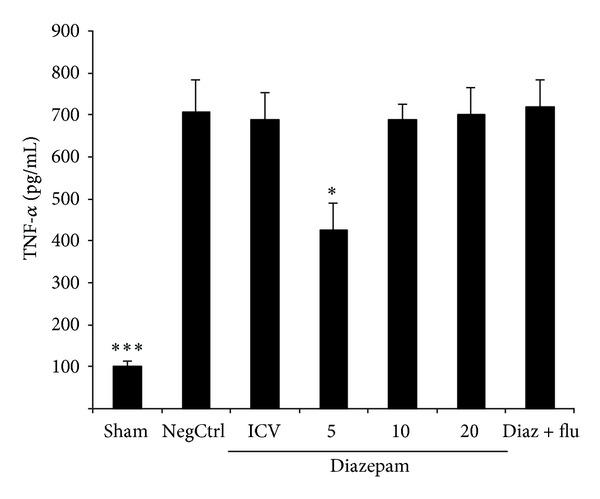
Effect of diazepam on pancreatic TNF-alpha level (pg/mL) of cerulein-induced acute pancreatitis in mice. Sham: normal mice treated with normal saline (5 mL/kg i.p. and 2 *μ*L i.c.v), NegCtrl: negative control, mice with pancreatitis treated with normal saline (5 mL/kg and 2 *μ*L i.c.v), Diazepam: treated mice (5, 10, 20, mg/kg i.p. and 10 *μ*g i.c.v), Diaz + flu: diazepam (20 mg/kg i.p.) + flumazenil (1 mg/kg i.p.). Data are shown as means ± SEM of 6 animals for each group. *P < 0.05, ***P < 0.001 versus negative control (ANOVA).

Cerulein administration increased significantly TNF-alpha formation in vehicle-treated mice (negative control). Pancreas levels of TNF-alpha were significantly reduced (*P* < 0.05) in cerulean-treated mice which had been pretreated with diazepam in dose of 5 mg/kg by i.p. injection (Figure 2).

### 3.3. Effects of Diazepam on MPO Activity

**Figure 3:**
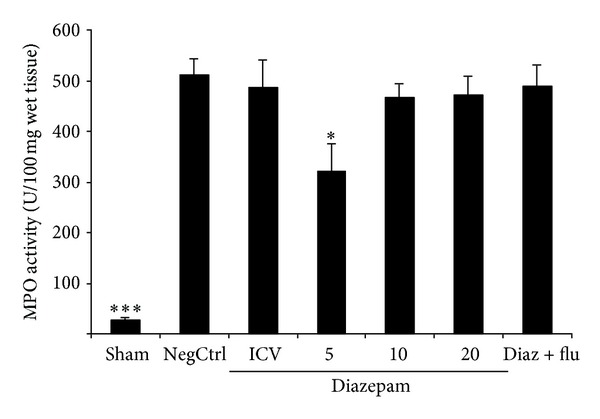
Effect of diazepam on pancreatic MPO (myeloperoxidase) activity (U/g wet tissue) of cerulein-induced acute pancreatitis in mice. Sham: normal mice treated with normal saline (5 mL/kg i.p. and 2 *μ*L i.c.v.), NegCtrl: negative control, mice with pancreatitis treated with normal saline (5 mL/kg i.p. and 2 *μ*L i.c.v.), Diazepam: treated mice (5, 10, 20 mg/kg i.p. and 10 *μ*g i.c.v.), Diaz + flu: diazepam (20 mg/kg i.p.) + flumazenil (1 mg/kg i.p.). Data are shown as means ± SEM of 6 animals for each group. *P < 0.05, ***P < 0.001 versus negative control (ANOVA).

MPO activity as a marker of leukocyte accumulation was obviously enhanced in the pancreas tissue following the cerulein administration. Pretreatment with diazepam at dose of 5 mg/kg by i.p. injection significantly reduced MPO activity, in comparison to saline treated mice (*P* < 0.05) (Figure 3).

### 3.4. Effects of Diazepam on the Histological Parameters

In normal saline treated mice, pancreas did not show any tissue injuries at light microscopic level (×10 magnification). Administration of cerulein induced acute edematous with severe leukocyte infiltration pancreatitis in all mice tested. Prominent interlobular and severe intralobular edema was also accompanied with moderate perivascular and abundant diffuse inflammatory infiltration. 

**Figure 4:**
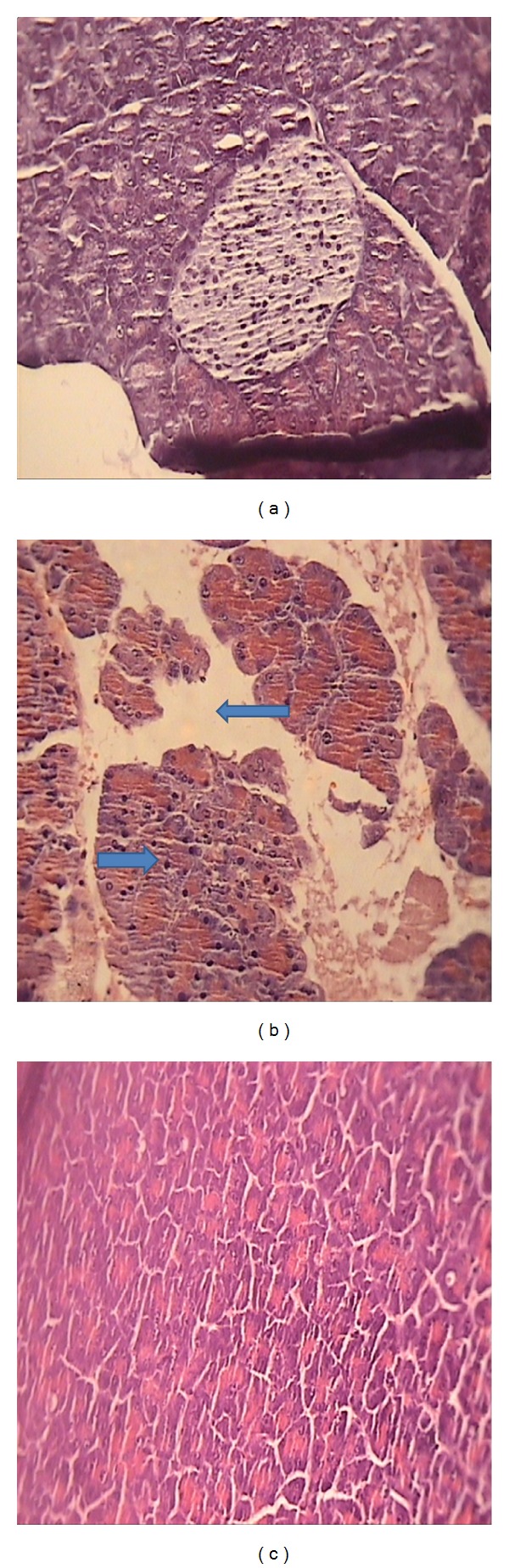
Representative illustration of normal pancreas and acute pancreatitis in mice. (a) Normal pancreatic tissue. (b) Acute pancreatitis induced by cerulein with severe intralobular edema and abundant diffuse infiltration. (c) Acute pancreatitis treated with diazepam (5 mg/kg i.p.) with moderate intralobular edema and scarce perivascular infiltration. H&E staining with low (×10) power.

In groups that received diazepam in the dose of 5 mg/kg i.p., the severity of edema and leukocyte infiltration was significantly reduced compared to normal saline treated group (*P* < 0.05) (Table 1, Figure 4).

## 4. Discussion

The present data showed that pretreatment with diazepam before the induction of acute pancreatitis decrease the susceptibility to the disease and histological lesions.

Our results demonstrated that administration of diazepam (5 mg/kg i.p.) reduced amylase and lipase activity, TNF-alpha, MPO activity, and pathological alterations, which are markers of pancreatitis. Action of diazepam on PBRs was already reported as being related to its effects on acute inflammation [20]. This is in accordance with the results obtained by Lazzarini et al. which demonstrated that the diazepam was effective to protect against carrageenan-induced acute inflammatory paw edema [11]. They suggested that the beneficial effect was related to an action of diazepam on the peripheral-type benzodiazepine receptor (PBR) present in the adrenal and/or immune/inflammatory cells. Also it has been found that treatment of mice with PBRs selective agonist, Ro5-4864, obviously decreases the ability of macrophages to produce interleukin-1, TNF-*α*, and interleukin-6 [14]. Current studies have been demonstrated that the development of pancreatitis is associated with expression of proinflammatory cytokine within the pancreas, increasing as pancreatitis severity increases, and the expression of IL-1*β*, and TNF-alpha mRNA concurs with the development of histologic lesions in pancreatitis and hyperamylasemia [21, 22].

So it could be assumed that some beneficial effects of diazepam (5 mg/kg i.p.) on pancreatitis are because of anti-inflammatory effects of diazepam which mediate through PBRs. Also in this study to make distinguish between the central and peripheral effects of diazepam, we evaluated the supraspinal effect of diazepam (i.c.v) in pancreatitis. As results showed anti-inflammatory effect of diazepam (i.c.v.) was not prominent so the beneficial effects of diazepam (5 mg/kg i.p.) could be solely attributed to its actions on PBRs. Administration of diazepam increases plasma level of corticosterone with plasma peak after 90 minute of injection, via acting on PBRs on adrenal glands [23, 24]. It has been shown that endogenous glucocorticoids are important factors for acinar cell survival which protects acinar cells by decreasing their sensitivity to the cell death during cerulein induced acute pancreatitis [13]. 

Despite our expectation, diazepam at higher test doses did not show any protection against cerulean-induced acute pancreatitis. It has been demonstrated that diazepam enhanced apoptosis in cancer cells in presence of cytotoxic agents, by mitochondrial transmembrane potential drop that facilitated release of apoptogenic factors such as cytochrome c and apoptosis-inducing factor in mitochondria [25, 26]. Cerulein through cholecystokinin receptors mediates the secretory and inflammatory events. Free oxygen radicals that generated from cerulein cause induction of oxidant sensitive transcription factor (nuclear factor kappa-light chain-enhancer of activated B cells, NF-*κ*B) activation, thereby enhance cytokine expression [27]. Also high concentration of cerulein promoted the expression of proapoptotic gene bax and p53 and DNA fragmentation [28]. According to our results it may be hypothesized that diazepam at higher doses probably enhanced apoptosis in pancreatic tissue following administration of cerulein.

For determination the exact mechanisms of diazepam and role of PBRs in the pathogenesis of pancreatitis, it should be better to investigate the effect of PBRs selective agonists such as Ro5-4864 or PK 11195 on the severity of experimental pancreatitis. Therefore, we suggest further researches using selective PBRs agonists. 

## 5. Conclusion

 Here we demonstrated that pretreatment with diazepam showed anti-inflammatory effects against cerulein-induced acute pancreatitis in mice probably via acts on peripheral benzodiazepine receptor. But further experiments with selective PBRs agonists need to determine the principle mechanisms of diazepam in this situation. 

## Figures and Tables

**Table 1:** Effect of diazepam administration on pathological scores of pancreas tissue in cerulein-induced acute pancreatitis in mice; Sham: normal mice treated with normal saline (5 mL/kg i.p. and 2 *µ*L i.c.v), NegCtrl: negative control treated with normal saline (5 mL/kg i.p. and 2 *µ*L i.c.v), Diazepam: treated mice (5, 10, 20 mg/kg i.p. and 10 *µ*g i.c.v.), Data are shown as means ± SEM, *n* = 6 (Mann-Whitney *U* test), *P < 0.05: significant difference compared to negative control group.

Group	Route	Edema	Leukocyte infiltration
Sham	i.p.	0.0	0.0
NegCtrl	i.p.	2.6 ± 0.2	2.6 ± 0.2
Diazepam (20 mg/kg i.p.)	i.p.	2.1 ± 0.3	2.3 ± 0.3
Diazepam (10 mg/kg i.p.)	i.p.	2.0 ± 0.3	2.1 ± 0.3
Diazepam (5 mg/kg i.p.)	i.p.	1.3 ± 0.2*	1.5 ± 0.2*
Diazepam (10 *µ*g/mouse ICV)	i.c.v	2.2 ± 0.3	2.1 ± 0.4
Diaz + flu (diazepam 20 mg/kg + flumazenil 1 mg/kg)	i.p.	2.3 ± 0.3	2.4 ± 0.6
